# Defining Optimal Timed Up and Go Test Cut-Offs for Fall Risk in Older Adults in Institutional Care in Malaysia

**DOI:** 10.21315/mjms-05-2025-367

**Published:** 2025-10-31

**Authors:** Zahira Zohari, Hakimah Sallehuddin, Azliza Rahim, Hazwan Mat Din, Nurfaizah Saibul, Asiah Jafri, Shafikah Rahim, Faizah Nasir, Sri Saliza Salleh, Wan Rabiatul Adawiah Wan Rosdi

**Affiliations:** 1 Geriatric Unit, Medical Department, Faculty of Medical and Health Sciences, Universiti Putra Malaysia, Serdang, Selangor, Malaysia; 2 Clinical Research Unit, Hospital Sultan Abdul Aziz Shah, Universiti Putra Malaysia, Serdang, Selangor, Malaysia; 3 Nursing Department, Hospital Sultan Abdul Aziz Shah, Universiti Putra Malaysia, Serdang, Selangor, Malaysia; 4 Malaysian Research Institute on Ageing (MyAgeing), Universiti Putra Malaysia, Serdang, Selangor, Malaysia

**Keywords:** falls, fall prevention, institutional care, long-term care, Timed Up and Go test

## Abstract

**Background:**

The Timed Up and Go (TUG) test effectively identifies risk of fall, especially in institutional care settings with limited resources. This is the first Malaysian study assessing the validity of the TUG test against the Downton Fall Risk Index (DFRI) and establishing an optimal TUG cut-off for institutional settings.

**Methods:**

This cross-sectional study included residents aged > 60 years who could ambulate independently with or without aid from Rumah Seri Kenangan Cheras. Demographic data, TUG test scores, and DFRI scores were collected for fall risk assessment. Receiver operating characteristic (ROC) analysis was used to assess the validity of TUG, with the area under the curve (AUC) measuring sensitivity and specificity, and the Youden Index identifying the optimal cut-off.

**Results:**

Of the 192 residents, 92 (47.9%) fulfilled the inclusion and exclusion criteria. The mean age was 72.24 years (standard deviation, SD = 8.42), and 33.7% were classified as having a high risk of falling (DFRI ≥ 3). The TUG test showed an AUC of 0.65 (95% Confidence Interval [CI]: 0.54–0.77, *P* = 0.018), indicating moderate validity. A cut-off of 12.5 s achieved high sensitivity (93.5%) and low specificity (34.4%).

**Conclusion:**

The TUG test cut-off point identified was 12.5 s, which is lower than the standard 13.5 s reported for community-dwelling older adults. This difference reflects the unique characteristics of institutionalised older adults, who often experience greater physical and cognitive impairments. Environmental factors and methodological variations may contribute to this discrepancy, emphasising the need to set specific cut-off points to assess risk of fall in this population accurately.

## Introduction

Falls in older adults pose a significant physical, psychological, and economic burden (1, 2). Globally, approximately 30% of older adults experience falls, and this rate doubles for older adults living in institutional care or nursing homes (3–5). According to a previous study, the prevalence of fall in a nursing home in Penang, Malaysia was approximately 32% (6), and another smaller study reported a 30% prevalence in Kuala Lumpur (7). Moreover, falls in institutional or long-term care settings represent a defeat, as these facilities are intended to provide care and safety for residents.

The World Falls Guidelines state that residents of institutional care are at a high risk of falls (8). Several factors contribute to an increased risk, including physical frailty, comorbidities, physical inactivity, polypharmacy, and unfamiliarity with new surroundings (9). Therefore, recognising and assessing the risk factors for falls in institutionalised care is crucial for developing effective fall prevention strategies and interventions.

Various tools have been developed to assess the risk of falls among older adults (10–12). No clear evidence exists regarding the best assessment tool for institutional or long-term care homes (13, 14). The tool must be user-friendly and capable of identifying individuals at a higher risk of falls.

The Timed Up and Go (TUG) test is a widely used fall risk assessment tool (15–17). To our knowledge, there is a paucity of global and local data on the TUG test cut-off for older adults residing in institutional care homes. A specific cut-off point for the TUG test in this population could serve as an important indicator for healthcare providers to focus their resources on individuals at a high risk of falls (18, 19).

This study aimed to identify the optimal cut-off points for the TUG test in an institutional setting to enable effective fall risk identification. We evaluated the agreement and validity of the TUG test using the Downton Fall Risk Index (DFRI), a widely used tool for assessing fall risk. The findings of this study provide valuable insights for healthcare professionals, administrators, and policymakers in developing evidence-based fall prevention strategies for older adults in long-term care settings.

## Methods

### Study Design and Participants

This cross-sectional study evaluated the criterion validity of the DFRI against the TUG test to identify the cut-off point for the TUG test among older adults in institutional care settings.

This study was conducted at Rumah Seri Kenangan Cheras (RSKC), a publicly funded institutional care facility in Selangor, Malaysia, which houses 192 residents. The inclusion criteria were participants aged 60 years and older who could ambulate independently, with or without the use of a walking aid. Individuals who were bedbound or unable to walk independently (e.g., reliant on a wheelchair or person-assisted mobility) were excluded from the study.

### Data Sources and Management

This study utilised data collected from fall risk assessments and medical records during a 12-month “Knowledge Transfer Project” titled “Program Pemerkasaan Perkhidmatan Penjagaan Kesihatan Warga Emas di Rumah Seri Kenangan Cheras, Selangor melalui Kepakaran Penjagaan Geriatrik (Empowerment Programme for Elderly Healthcare Services at RSKC, Selangor through Geriatric Care Expertise),” conducted between February 2023 and March 2024. This project aimed to strengthen geriatric care services at the facility by integrating routine assessments and tailored health interventions. It involves a multidisciplinary team comprising specialists in geriatrics, internal medicine, psychiatry, physiotherapy, nutrition, nursing, and research.

Data collection involved extracting information from standardised fall risk assessment sessions conducted by physiotherapists and nurses. The DFRI and TUG tests were conducted during the same assessment session to ensure consistency in timing. However, in some instances, they were performed on different days owing to operational constraints, such as staff availability, the resident’s medical condition, or scheduling logistics. All assessments were conducted within the same admission period to ensure their clinical relevance. Additional demographic and clinical data were obtained from medical records.

#### DFRI

The DFRI is a validated screening instrument recommended for use in hospitals, geriatric clinics, long-term care facilities for older adults, and primary care settings (20–22). There were 11 risk items, each scoring one point. A score of ≥ 3 indicates a high risk of falls (Supplementary Table 1). The risks include a history of falls, medication use, sensory deficits, mental state, and gait. Several studies have shown that the DFRI can predict falls in residential care (20, 23).

#### TUG

The TUG test is a timed assessment of functional mobility, in which the participant stands up from a standard armchair, walks to a line on the floor 3 m away, turns around, walks back to the chair, and sits down (17, 18). The cut-off point was the value at which the TUG test indicated an increased risk of falling. TUG values ranging from 10 s to 25 s can distinguish between individuals who have experienced falls and those who have not (19, 24–26).

### Sample Size and Sampling Method

The sample size was determined by calculating the receiver operating characteristic (ROC) analysis principle using MedCalc Statistical Software (MedCalc Software Ltd., Ostend, Belgium), with a Type I error rate of 0.05, a study power of 80%, and an expected area under the curve (AUC) of 0.70. Based on these parameters, the minimum sample size required was 62 participants (27, 28).

Purposive sampling was conducted. All RSKC residents were screened based on the inclusion and exclusion criteria through a comprehensive review of their medical records. Participants with incomplete TUG or DFRI data were excluded from the analysis. No imputation was performed for missing values. Data quality assurance was ensured by cross-checking the data for consistency and accuracy. All data were anonymised to ensure privacy and confidentiality.

### Statistical Method

Descriptive statistics are reported as mean (standard deviation, SD) for continuous variables and frequency (percentage) for categorical variables. Spearman’s rank-order correlation was used to assess the relationship between TUG and DFRI total scores.

ROC curve analysis was used to evaluate the criterion validity of the TUG test, using the DFRI as the reference standard. AUC was calculated to determine the overall discriminatory ability of the test (35). The Youden Index (YI) was used to identify the optimal cut-off point for the TUG test. The YI was calculated for each coordinate of the ROC curve, and the cut-off point with the highest YI value was selected as the optimal threshold, reflecting the best combination of sensitivity and specificity. All data were analysed using SPSS version 23 (IBM Corp., Armonk, NY, USA).

## Results

A total of 92 of the 192 (47.9%) residents of Rumah Seri Kenangan fulfilled the inclusion criteria. Their mean (SD) age was 72.24 years (8.42), and 55.4% were male. Malay participants accounted for 64.1% of the total, and 48.9% of the participants had hypertension. According to the DFRI, 33.7% of patients were classified as having a high risk of falls, whereas 21.7% had a history of falls in the past year. The mean (SD) TUG time was 20.04 (13.24) s. The participants’ characteristics are presented in Table 1.

**Figure 1:**
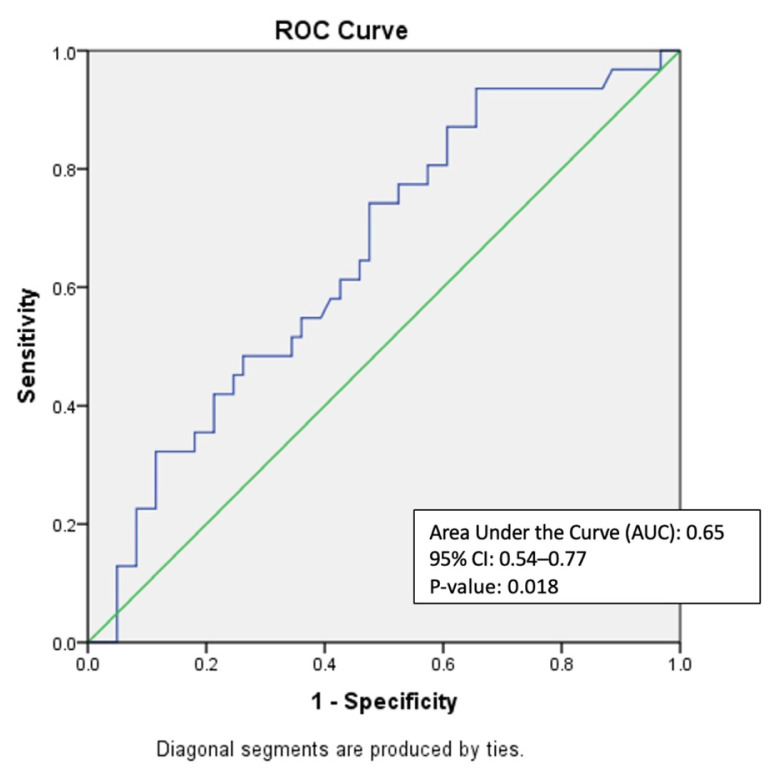
ROC curve for TUG test Blue line = The diagnostic performance of the TUG test; Green line = The line of no discrimination (AUC = 0.50), indicates a test with no diagnostic ability; The ROC curve demonstrates the relationship between sensitivity (true positive rate) and 1-specificity (false positive rate) for the TUG test; AUC: 0.65, showing moderate diagnostic capability (95% CI: 0.54–0.77, *P* = 0.018)

Figure 1 shows the ROC curve used to assess the criterion validity of the TUG test using the DFRI as the reference. The AUC was 0.65 (95% CI: 0.54–0.77, *P* = 0.018), indicating a moderate discriminatory ability of the TUG test in distinguishing between participants with high and low fall risk based on the DFRI.

Spearman’s rank-order correlation was used to assess the relationship between the TUG and DFRI total scores. A weak, positive correlation was statistically significant, r_s_(58) = 0.27, *P* = 0.027. This finding suggests a modest association between the two fall risk measures.

Table 2 presents various TUG score cut-off values and their corresponding sensitivities, specificities, and YI. The YI was used to identify the optimal cut-off point. The optimal TUG cut-off value was identified as 12.5 s, based on its high sensitivity (93.5%) and the highest YI (0.279) among all tested thresholds. Although the false positive rate at this cut-off was relatively high (65.6%), it enabled the detection of most individuals who were truly at high risk of falls. The results of the diagnostic performance of the TUG score cut-off value of 12.50 s against the DFRI high risk of falls are presented in Table 3.

## Discussion

The study included residents with a mean age of 72.2 years, 33.7% of whom were categorised as having a high risk of falls according to the DFRI. All participants were independently mobile, although more than one-fifth had a history of recent falls. These characteristics reflect a functionally capable yet clinically vulnerable population, emphasising the need for sensitive screening tools, such as the TUG test, in institutional care.

We acknowledge the availability of other validated and reliable tools for assessing fall risk among older adults, such as the STRATIFY, Morse Fall Scale, and Berg Balance Scale (29–31). However, the DFRI was selected as the reference standard in this study because it offers a more holistic assessment aligned with the multifactorial nature of fall risk in institutional settings. The DFRI evaluates cognitive status, sensory impairments, mobility, medication use, and comorbidities, which are key domains often associated with falls among older adults. This “one-stop” assessment approach makes it especially suitable for populations in long-term care facilities, where fall risk is rarely attributed to a single factor (20–21, 32).

Rosendahl et al. (20) reported that the DFRI helps predict falls in older adults living in residential care facilities, particularly within the first 3 months of assessment. They reported that individuals in the high risk group (DFRI score ≥ 3) had a 36% higher risk of falling than those in the low-risk group. This supports the use of the DFRI as a valid comparator in studies assessing fall risk in institutionalised settings (34).

Therefore, rather than replacing comprehensive tools, the TUG test may be a useful complementary tool. Its rapid administration can flag individuals for further evaluation using more detailed multi-domain tools, making it an efficient component of tiered fall risk assessment strategies in institutional settings.

### Balancing Sensitivity and Specificity in Fall Risk Assessments

One of the main considerations in selecting an optimal cut-off point for the TUG test is to achieve an appropriate balance between sensitivity and specificity. Sensitivity reflects the ability of the test to correctly identify individuals at high risk of falls, whereas specificity reflects the ability to classify those who are not at risk correctly. In this study, the chosen cut-off point of 12.5 s provided a high sensitivity of 93.5%, indicating that most individuals at high risk of falls were identified. However, this is associated with a 65.6% false positive rate (i.e., low specificity), indicating that some individuals who are not at risk may be misclassified.

Although an AUC of 0.65 falls below the cited threshold of 0.7 for strong validity, it still reflects a statistically significant (*P* = 0.018) and fair discriminatory ability to differentiate fall risk among the study population. We acknowledge this limitation and have interpreted it within the context of institutional care settings, where fall prevention is a high priority. In resource-limited settings, even a modestly accurate but statistically significant tool can have substantial clinical value, particularly when the screening tool is simple, time-efficient, and easy to administer by non-specialist staff. In this population, prioritising sensitivity is essential, as missed cases may result in serious outcomes, such as fall-related hip fractures, hospitalisation, or long-term functional decline (5, 8, 26). This is consistent with previous literature, where AUC values between 0.6 and 0.7 are considered acceptable for screening purposes when clinical relevance and feasibility are high (34).

We calculated the YI (sensitivity + specificity −1) for each cut-off to determine the most appropriate threshold (40, 41). This index identifies the point that offers the best overall balance between sensitivity and specificity. Among the thresholds tested, 12.5 s yielded the highest YI (0.279), supporting it as the most balanced and effective cut-off.

At the identified TUG cut-off point of 12.50 s, the test demonstrated high sensitivity (93.5%) and a high negative predictive value (NPV) (91.3%), indicating that it is effective in identifying individuals who are not at a high risk of falls. This is valuable in institutional care settings, where missed cases can have severe consequences. However, the low specificity (34.4%) and modest positive predictive value (PPV) (42.0%) reflected a tendency to generate false positives, indicating that some residents may be classified as high risk despite not meeting the threshold on a multifactorial assessment. The likelihood ratios (LR^+^ = 1.43, LR^−^ = 0.19) further support this, as the low LR^−^ strengthens its role in ruling out risk, whereas the low LR^+^ suggests limited stand-alone diagnostic value. Collectively, these findings reinforce the utility of TUG as a complementary screening tool rather than a replacement for comprehensive fall risk assessments such as the DFRI.

Several studies have proposed different TUG test cut-off points for identifying fall risk, particularly among community-dwelling older adults. For instance, Shumway-Cook et al. (19) reported a widely cited threshold of 13.5 s while systematic reviews by Barry et al. (18) suggested a range between 10 s and 13.5 s. These thresholds were derived from ambulatory, community-dwelling adults and may not fully reflect the functional limitations present in institutionalised settings. Other studies have reported cut-off times between 12 s and 15 s, yielding sensitivities of 77% to 93% and specificities of 61% to 87% (15, 18).

The mean TUG test time in this cohort was 20.04 s, which was considerably higher than the identified cut-off of 12.5 s. This suggests that a substantial portion of the institutionalised population, although independently mobile, exhibits slower mobility. This supports the argument that even ambulatory older adults in institutional care may have functional impairments that place them at a greater risk of falling.

### The Importance of an Easy Fall Risk Assessment in Resource-Limited Settings

In institutional or long-term care settings, where staffing levels are low and staff may lack specialised training, a simple yet effective fall risk screening tool is crucial. The TUG test requires minimal resources and training, making it an ideal tool for these environments. Its simplicity allows staff to quickly assess the mobility of older adults and identify those at a high risk of falling. In facilities with constrained resources, conducting detailed and complex assessments may not be feasible, and the time required for comprehensive evaluations may be limited. This can empower staff members to take proactive measures to reduce falls, especially in facilities where specialised geriatric expertise may not be readily available.

This study only included individuals with independent mobility (with or without walking aids) despite living in institutional care due to social factors. This explains the TUG cut-off point being similar to that of community-dwelling older adults (12.5 s versus 10 s to 13.5 s). Therefore, the results of this study indicate that, although these individuals were independent of mobility, they required adequate resources to prevent falling. Annual screening using the TUG test can help stakeholders identify the necessary resources (staff, environmental modifications, and multicomponent programmes) for implementing fall prevention and intervention in each institutional care setting.

### Variability of TUG Cut-off

Regional and cultural differences may significantly influence TUG test cut-off values. Factors such as healthcare infrastructure, environmental conditions, and access to preventive resources may influence the overall fall risk in different regions. For example, regions with greater access to rehabilitation services and mobility aids may have a lower baseline fall risk than those with limited access. Environmental factors, such as crowded living conditions, uneven flooring, and limited indoor space, may impact residents’ mobility and TUG test performance in institutional care settings (37, 38).

Cultural attitudes toward ageing, mobility, and fall prevention play crucial roles. In rural regions with limited education levels, ageism, and low awareness, older adults may engage in fewer independent activities, potentially reducing their exposure to fall risks and limiting their mobility (39). Conversely, in cultures that encourage independence, there may be a higher prevalence of fall incidents owing to increased movement and self-reliance, which may affect the observed cut-off values (40, 41)

These variations underscore the importance of tailoring TUG test cut-off values to reflect local and cultural contexts, thereby ensuring the accuracy and applicability of fall risk assessments across diverse populations.

### Limitations

This study had certain limitations that warrant consideration. First, the sample size was calculated based on an anticipated AUC of 0.70, whereas the observed AUC was 0.65. This deviation from the expected value may have reduced the power of the study.

The AUC of 0.65 indicates only fair discriminative ability. Although this may not meet the conventional thresholds for strong diagnostic accuracy, it is still considered clinically acceptable in settings where ease of administration and high sensitivity are prioritised. A larger sample size across multiple institutions could strengthen the validity of the identified TUG cut-off point for fall risk in institutionalised older adults.

Moreover, recall bias may have affected the accuracy of the data on fall history. As fall histories were self-reported or reported by staff, there may have been inaccuracies or underreporting. This bias could have affected the correlation between the TUG scores and actual fall risk.

Additionally, the study was conducted in a single facility, and the care homes varied widely in terms of resident characteristics. Differences in resident age, health conditions, and levels of dependency between facilities could influence fall risk and, consequently, the applicability of the TUG test’s cut-off point. Environmental and infrastructural differences across nursing homes, such as room layout, flooring, lighting, and available mobility aids, affect residents’ mobility and fall risk. These variations suggest that a TUG cut-off validated in one facility may not be directly applicable to others with different conditions.

## Conclusion

This study identified a specific cut-off point for institutional and long-term care settings, providing valuable data to assist healthcare professionals in making more informed decisions regarding fall prevention strategies. Long-term care staff can better identify high risk individuals, thereby enabling tailored interventions. The diversity in the cut-off points highlights the need for ongoing research and validation in various environments. This approach will ensure that fall risk assessments are appropriately adapted to various groups of older adults, ultimately enhancing their overall safety and quality of life in institutional care settings.

## Supplementary Information

**Supplementary Table 1:** Criteria in Downtown Falls Risk Index

Operational Term	Definition
Fall	A fall is definedas an event that results in a person coming to rest inadvertently on the ground or floor or other lower level (47)
History of falls	Known history of falls during the preceding year
Medications	All medications which are currently taken by the residents including: - Tranquilisers/Sedatives - Anti-hypertensives - Diuretics - Anti-parkinsonian - Anti-depressants - Other medications
Sensory deficit	Sensory deficits include the presence of: - Visual impairment: subject was not able to read a short text in 10-mm block letters at a reading distance - Hearing impairment: unable to perceive a conversation in a normal voice at a distance of 1 m - Limb impairment: defined as the presence of amputated limbs or signs of extremity paresis
Mental state	Mental state is defined by orientated or confused (impaired cognition)
Gait	- Subjects were classified as “unable” if they were described as unable to walk in the physiotherapy team’s documentation, even with physical assistance - Subjects were classified as “unsafe with/without aids” if the team noted that physical assistance or supervision was required for walking - Subjects were classified as “safe/normal” (i.e., safe with or without aids) if the physiotherapy team judged that no such precautions were necessary

## Figures and Tables

**Table 1:** Participant’s characteristics

Characteristic	Frequency, *n* (*N* = 92)	Percentage, %
Age, mean ± SD	72.24 ± 8.42	
Gender		
Male	51	55.4
Female	41	44.6
Ethnicity		
Malay	59	64.1
Chinese	18	19.6
Indian	15	16.3
Comorbidities		
Hypertension	45	48.9
Diabetes Mellitus	21	22.8
Ischaemic Heart Disease	12	13.0
Hyperlipidaemia	9	9.8
Psychiatric Illness	10	10.8
Asthma	10	10.8
Polypharmacy		
Yes	23	25.0
No	69	75.0
History of fall		
Yes	20	21.7
No	72	78.3
High Risk of Fall (based on DFRI)		
Yes	31	33.7
No	61	66.3
TUG Time (sec), mean ± SD	20.04 ± 13.24	

**Table 2:** Cut-off value determination of TUG score for high fall risk using DFRI as the reference

Cut-off value of TUG score (sec)	Sensitivity (%)	1-Specificity (%)	Specificity (%)	Youden Index
29.44	22.6	8.2	91.8	0.144
26.63	32.2	11.5	88.5	0.207
22.54	41.9	21.3	78.7	0.206
20.63	48.4	26.2	73.8	0.222
15.13	67.7	47.5	52.5	0.202
**12.50**	93.5	65.6	34.4	**0.279**

Youden Index = Sensitivity + Specificity − 1

**Table 3:** Diagnostic performance for the TUG cut-off value of 12.50 seconds against the DFRI

		DFRI High risk of fall, *n*		
		Yes	No	
**TUG high risk of fall**	Yes	29 (TP)	40 (FP)	PPV = 42.0%
	No	2 (FN)	21 (TN)	NPV = 91.3%
		Sn = 93.5%)	Sp = 34.4%	

TP = true positive; FP = false positive; FN = false negative; TN = true negative; PPV = positive predictive value; NPV = negative predictive value; Sn = sensitivity; Sp = specificity
